# Embedded Sensor Data Fusion and TinyML for Real-Time Remaining Useful Life Estimation of UAV Li Polymer Batteries

**DOI:** 10.3390/s25123810

**Published:** 2025-06-18

**Authors:** Jutarut Chaoraingern, Arjin Numsomran

**Affiliations:** 1 Department of Robotics and AI Engineering, School of Engineering, King Mongkut’s Institute of Technology Ladkrabang, Bangkok 10520, Thailand; jutarut.ch@kmitl.ac.th; 2 Department of Instrumentation and Control Engineering, School of Engineering, King Mongkut’s Institute of Technology Ladkrabang, Bangkok 10520, Thailand

**Keywords:** real-time embedded systems, remaining useful life (RUL) estimation, sensor data fusion, TinyML, UAV Li polymer battery monitoring

## Abstract

The accurate real-time estimation of the remaining useful life (RUL) of lithium-polymer (LiPo) batteries is a critical enabler for ensuring the safety, reliability, and operational efficiency of unmanned aerial vehicles (UAVs). Nevertheless, achieving such prognostics on resource-constrained embedded platforms remains a considerable technical challenge. This study proposes an end-to-end TinyML-based framework that integrates embedded sensor data fusion with an optimized feedforward neural network (FFNN) model for efficient RUL estimation under strict hardware limitations. The system collects voltage, discharge time, and capacity measurements through a lightweight data fusion pipeline and leverages the Edge Impulse platform with the EON™Compiler for model optimization. The trained model is deployed on a dual-core ARM Cortex-M0+ Raspberry Pi RP2040 microcontroller, communicating wirelessly with a LabVIEW-based visualization system for real-time monitoring. Experimental validation on an 80-gram UAV equipped with a 1100 mAh LiPo battery demonstrates a mean absolute error (*MAE*) of 3.46 cycles and a root mean squared error (*RMSE*) of 3.75 cycles. Model testing results show an overall accuracy of 98.82%, with a mean squared error (*MSE*) of 55.68, a mean absolute error (*MAE*) of 5.38, and a variance score of 0.99, indicating strong regression precision and robustness. Furthermore, the quantized (int8) version of the model achieves an inference latency of 2 ms, with memory utilization of only 1.2 KB RAM and 11 KB flash, confirming its suitability for real-time deployment on resource-constrained embedded devices. Overall, the proposed framework effectively demonstrates the feasibility of combining embedded sensor data fusion and TinyML to enable accurate, low-latency, and resource-efficient real-time RUL estimation for UAV battery health management.

## 1. Introduction

Unmanned aerial vehicles (UAVs) have evolved into versatile platforms employed across a broad range of applications, including aerial photography, surveillance, parcel delivery, and infrastructure inspection. Among the various factors influencing UAV performance, battery life remains a principal constraint that limits operational duration and mission reliability. Accurate estimation of the remaining useful life (RUL) of lithium-polymer (LiPo) batteries is essential to enhance flight safety, mitigate the risk of in-flight power failure, and enable more effective task scheduling and route optimization. Furthermore, reliable RUL prediction facilitates predictive maintenance strategies, thereby extending battery service life and reducing replacement costs [1].

RUL estimation refers to the process of forecasting the time span from the current operational state until the point at which the system’s degradation exceeds acceptable thresholds under nominal operating conditions. Two primary approaches have been widely adopted for this task [2]. The first is the model-based approach, which relies on predefined physical or electrochemical models and utilizes sensor data to describe degradation behavior. The second is the data-driven approach, which leverages historical data and machine learning algorithms to predict degradation trends. In recent years, the data-driven paradigm has garnered increasing attention due to its flexibility and reduced dependence on explicit physical models, making it more adaptable to varying operating conditions and system types [3].

Several data-driven approaches for predicting the remaining useful life (RUL) of systems and components have been proposed in the literature. For example, ref. [4] employed a linear regression model to derive a health index (HI) from multidimensional sensor data to forecast the RUL of electrical machines. In ref. [5], a novel inheritance-based particle filter (PF) was introduced for RUL estimation of lithium-ion batteries, incorporating a Lamarckian inheritance mechanism within a genetic algorithm framework. This method demonstrated improved prediction accuracy while requiring fewer tuning parameters compared to traditional elitism-based genetic algorithm particle filters. Furthermore, ref. [6] proposed an enhanced RUL prediction technique utilizing an extended Kalman particle filter in combination with a modified double exponential degradation model, effectively addressing issues related to particle degeneracy in conventional PF algorithms. In another study, ref. [7] presented an online state-of-health (SOH) estimation method for lithium-ion batteries using support vector machines (SVMs) with radial basis function kernels, which relied on features extracted from partial charging voltage and current profiles.

Recent advancements in deep learning and artificial intelligence (AI) have enabled the development of sophisticated data-driven approaches for remain useful life (RUL) prediction. Deep neural networks (DNNs), which consist of multi-layer artificial neural architectures, are particularly effective for modeling complex nonlinear relationships and have demonstrated superior predictive performance in RUL estimation tasks. For instance, ref. [8] proposed a prognostics method based on deep convolutional neural networks (DCNNs), which effectively capture complex degradation features from sensor data, thereby enabling accurate RUL predictions that support enhanced maintenance planning and system reliability. Similarly, ref. [9] introduced a novel approach employing deep multi-scale convolutional neural networks to identify degradation patterns across multiple feature resolutions, leading to more precise and robust RUL forecasts. In another study, Wang et al. [10] utilized functional data analysis (FDA) to model degradation behavior captured in sensor readings, offering a framework capable of generating highly accurate RUL predictions and facilitating informed maintenance decision-making. Moreover, Ciani et al. [11] proposed an early-stage state-of-health (SOH) estimation method tailored for lithium batteries in wireless sensor networks. By leveraging long short-term memory (LSTM) networks in conjunction with a single exponential degradation model, their work demonstrates that accurate SOH prediction can be achieved with limited training data, thus significantly reducing the data requirements typically associated with reliable battery health forecasting.

Despite the promising performance of deep learning-based RUL estimation systems, several limitations hinder their practical deployment in real-world applications. These include high hardware infrastructure costs, elevated energy consumption, concerns regarding data privacy and security, limited network bandwidth, and significant latency when relying on cloud-based AI processing [12,13,14]. To address these challenges, various hybrid and resource-efficient solutions have been proposed. For instance, ref. [15] presented a deep learning framework combining convolutional neural networks (CNNs) and long short-term memory (LSTM) networks to improve RUL prediction accuracy in industrial systems. Using the NASA turbofan engine dataset, their approach outperformed both traditional machine learning algorithms and state-of-the-art methods in terms of reliability and prediction robustness for IoT device health monitoring. In the context of electric vehicle battery management, ref. [16] proposed a deep learning-based estimator capable of accurately determining battery capacity in real time. The model, trained using a mix of synthetic and real battery data, was validated within an Amesim-based EV powertrain simulation environment, demonstrating its applicability for real-time onboard diagnostics. Furthermore, ref. [17] introduced a cloud–edge-integrated approach utilizing lightweight temporal convolutional networks (LTCNs) to enhance RUL prediction accuracy while minimizing computational overhead. By deploying the model across both edge and cloud planes, the framework effectively balances performance with resource efficiency, making it a viable solution for latency-sensitive industrial prognostics.

**Figure 1:**
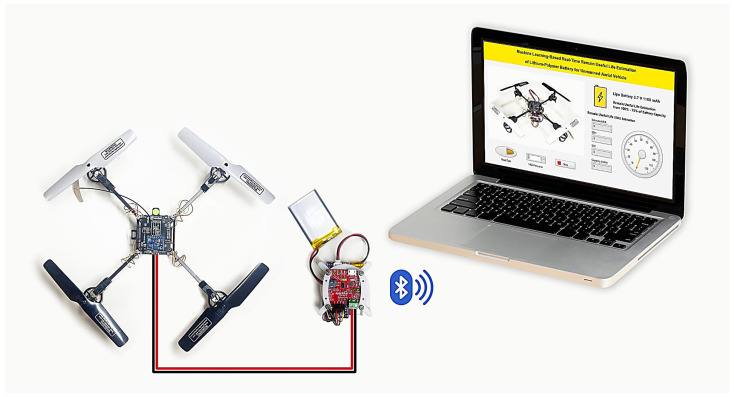
UAV, RUL LiPo battery-embedded AI sensor, and LabView application.

Although considerable progress has been made in the domain of UAV battery management, several critical challenges remain unresolved, including the high cost of hardware infrastructure and the limited computational capabilities of embedded systems. To overcome these limitations, this study introduces an end-to-end TinyML-based framework for real-time estimation of the remaining useful life (RUL) of lithium-polymer batteries deployed in UAVs, as depicted in Figure 1. The proposed solution is designed to achieve high predictive accuracy and low inference latency, while operating efficiently within the constraints of low-power, resource-limited embedded platforms.

The main contributions of this work are as follows:

- Embedded Sensor Data Fusion: A lightweight data fusion pipeline is developed to integrate voltage, discharge time, and capacity measurements into a unified feature set for the feedforward neural network (FFNN) model.
- TinyML Deployment: A compact RUL estimation model is implemented on the Raspberry Pi RP2040 microcontroller, enabling low-cost, real-world deployment.
- Model Optimization with Edge Impulse: The EON™Compiler is utilized to compress and optimize the neural network, enabling fast and energy-efficient on-device inference.
- Real-Time Monitoring: A LabVIEW application based on a state machine architecture is developed to visualize and monitor RUL predictions in real time.

This framework demonstrates that embedded sensor data fusion combined with TinyML can deliver high-accuracy, low-overhead RUL estimation, paving the way for safer, more reliable, and more efficient UAV battery management.

The remainder of this paper is structured as follows. Section 2 presents the materials and methods employed in this study, encompassing data acquisition, preprocessing, model training, and optimization. Section 3 introduces the embedded AI sensor system designed for real-time RUL estimation of UAV batteries, with emphasis on both hardware implementation and software architecture. Section 4 reports the experimental results and provides a detailed analysis of the system’s performance under real-world UAV operating conditions. Finally, Section 5 concludes the paper and highlights potential avenues for future research and system enhancement.

## 2. Materials and Methods

**Figure 2:**
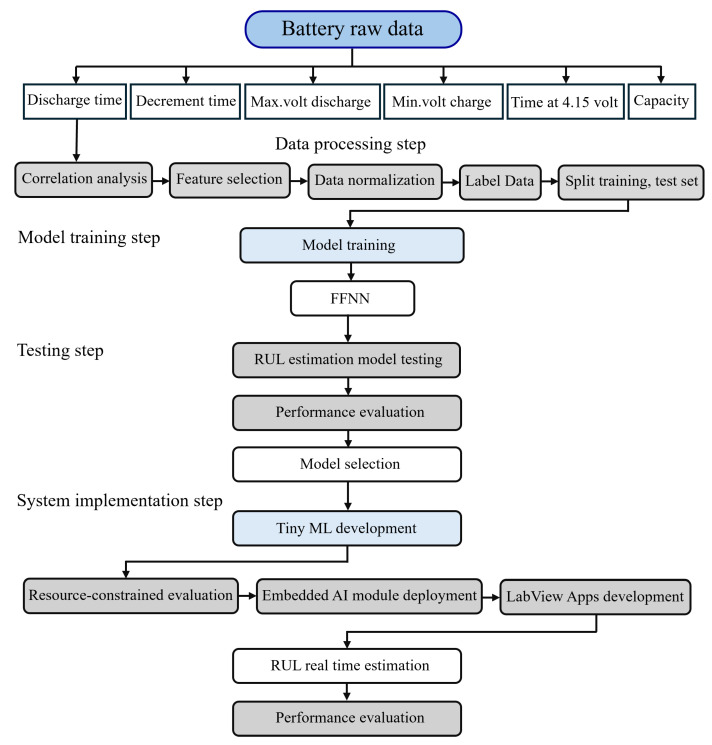
The proposed real-time RUL estimation structure.

The overall structure of the real-time remaining useful life (RUL) estimation framework for the battery system is illustrated in Figure 2. The process begins with the analysis of raw battery data, which are subsequently processed through a structured pipeline comprising correlation analysis, feature selection, normalization, data labeling, and partitioning into training and testing datasets. During the model training phase, feedforward neural networks (FFNNs), a type of machine learning algorithm, are applied to learn degradation patterns from the selected features. The testing phase evaluates the performance of the model and quantifies its prediction accuracy and reliability.

In the system implementation phase, the FFNN model is optimized using TinyML techniques for deployment on resource-constrained embedded hardware. The optimized model is integrated with an embedded AI sensor and connected to a LabVIEW-based application for real-time RUL estimation and system performance monitoring. This systematic methodology emphasizes effective model development and practical deployment for accurate RUL estimation in battery monitoring applications. The following subsections describe the methodology in four key stages: data acquisition, data preprocessing, model training and optimization, and model performance evaluation.

### 2.1. Data Acquisition

This study utilizes a dataset of lithium-polymer (LiPo) batteries (model LP-503562-IS-3, nominal voltage 3.7 V, capacity 1100 mAh) published by Galeotti et al. [18], which provides comprehensive capacity and electrochemical impedance data acquired under various states of charge (SOCs) and states of health (SOHs). Each battery was subjected to hundreds of standard and stress charge–discharge cycles. Key measurements include voltage, current, capacity, impedance spectra, and fitted equivalent circuit model (ECM) parameters. The capacity measurements were performed during full charge–discharge–charge cycles under controlled conditions (25 °C), while impedance spectra were acquired through galvanostatic electrochemical impedance spectroscopy (EIS) across 45 frequency points ranging from 0.2 Hz to 5000 Hz. All data were organized across three stages: standard cycling, partial discharge with EIS, and stress discharge for accelerated aging.

For this work, the dataset corresponding to two of the batteries was selected, comprising a total of 424 charge–discharge cycles. The data were labeled with cycle-level remaining useful life (RUL) values and used to train and evaluate multiple machine learning models. Although the dataset size may be considered moderate, it provides sufficient resolution to model battery degradation trends and to validate embedded RUL estimation frameworks. Cross-validation and hold-out methods were employed during model development to ensure robustness and generalizability. Table 1 summarizes the main characteristics of the charge and discharge protocols applied to the LiPo batteries used in the degradation dataset. The charging process followed a constant current–constant voltage (CC–CV) strategy, while the discharging process was conducted under constant current (CC) conditions with specific cut-off voltage thresholds.

Charge–discharge cycles were conducted iteratively until the measured capacity, denoted as *C*, diminished to less than 70% of the initial nominal capacity, represented as $C_{N}$. A capacity is determined by integrating the current over the discharge duration:

(1) $$C=\int_{0}^{t}I\left(t\right)\;dt,$$

state of charge is estimated relative to the initial capacity (*C* at stage 0), accounting for battery aging and capacity degradation [19]:

(2) $$SOC=1-\frac{1}{C}\int_{0}^{t}I\left(t\right)\;dt,$$

Battery RUL is the estimated remaining operational cycles ($M_{\mathrm{EOL}}$) minus the completed charge cycles ($M_{cc\alpha }$). In the dataset collection phase, the value of $M_{\mathrm{EOL}}$ can be obtained through direct measurement. When complete capacity versus cycle data is available up to the point of battery failure, the end-of-life cycle count is empirically defined as the cycle at which the battery capacity drops below a predetermined threshold, typically 70% of its nominal capacity. This allows the actual $M_{\mathrm{EOL}}$ to be explicitly determined from the experimental data, enabling accurate calculation of the remaining useful life (RUL) for any cycle α [20]:

(3) $${\mathrm{RUL}}_{\alpha }=M_{\mathrm{EOL}}-M_{cc\alpha },$$

**Figure 3:**
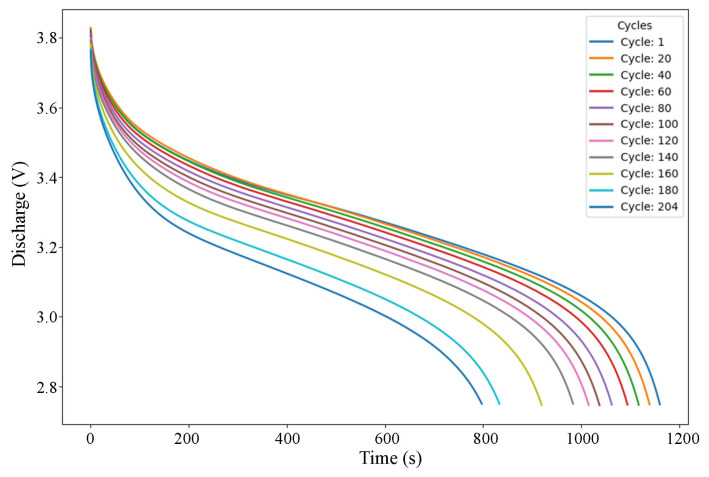
Discharge voltages’ (V) degradation trends.

**Figure 4:**
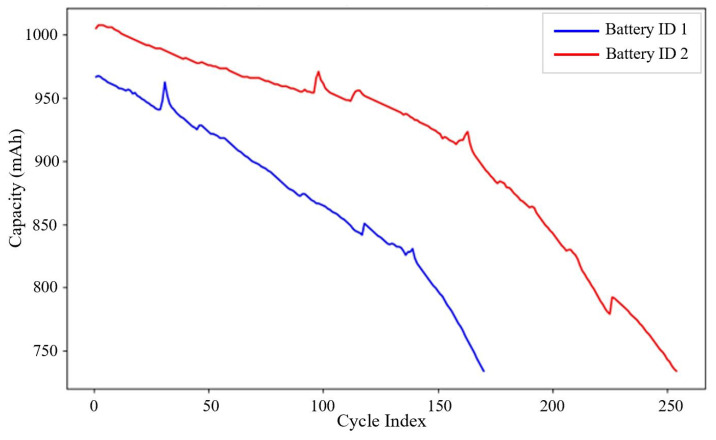
Capacity (mAh) degradation trends.

The capacity degradation trends for all batteries under standard discharge conditions are illustrated in Figure 3, which plots capacity against cycle numbers. The relationship between the cycle index and battery capacity (mAh) for two batteries, Battery ID 1 and 2, is depicted in Figure 4.

**Figure 5:**
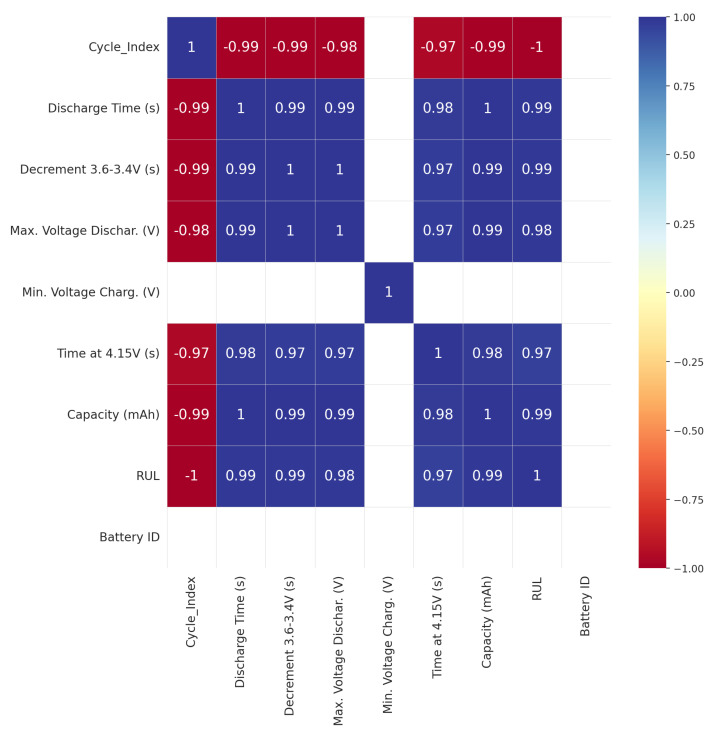
Remaining useful life (RUL) and other battery-related features.

Features that are derived from raw voltage, capacity, and time data over numerous charge–discharge cycles are valuable for predicting the remaining useful life (RUL) of the batteries, serving as key indicators of battery performance, and offering valuable insights into battery behavior, as indicated by the dataset. Table 2 provides a detailed view of the central tendencies (mean, median), variability (standard deviation), and ranges (min, max) for each feature, giving insight into the behavior of the batteries across multiple cycles. The correlation analysis presented in Figure 5 provided a heatmap of associations between RUL (remaining useful life) and other battery-related features. The heatmap reveals that RUL is most strongly and positively correlated with features that reflect the battery’s operational performance, such as discharge time, voltage decrement times, and capacity.

**Figure 6:**
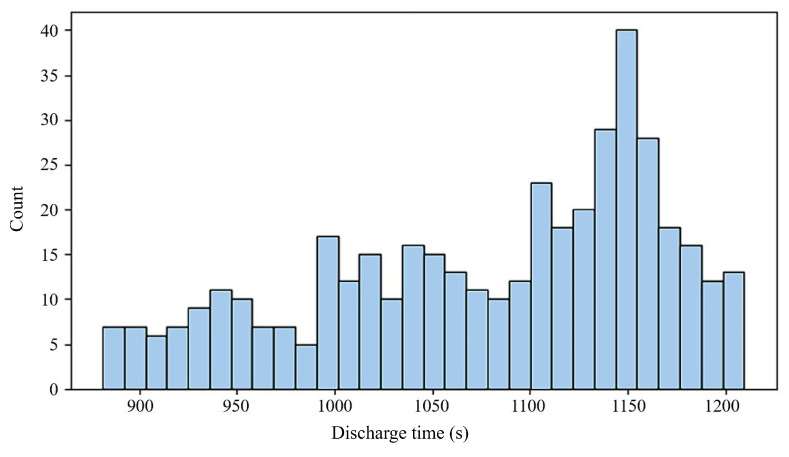
RUL-related feature histograms: discharge time (s).

**Figure 7:**
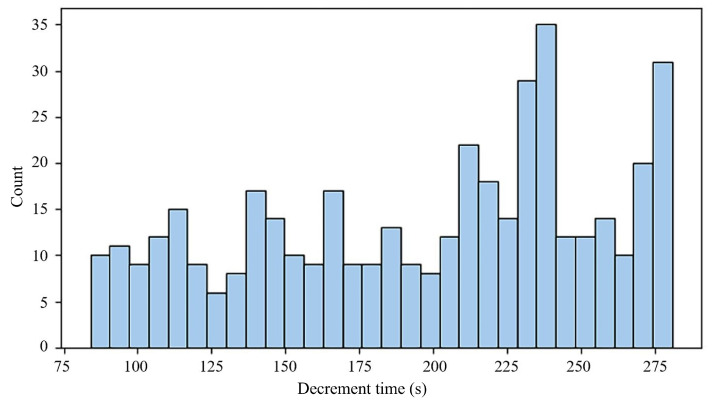
RUL-related feature histograms: decrement 3.6–3.4 V (s).

**Figure 8:**
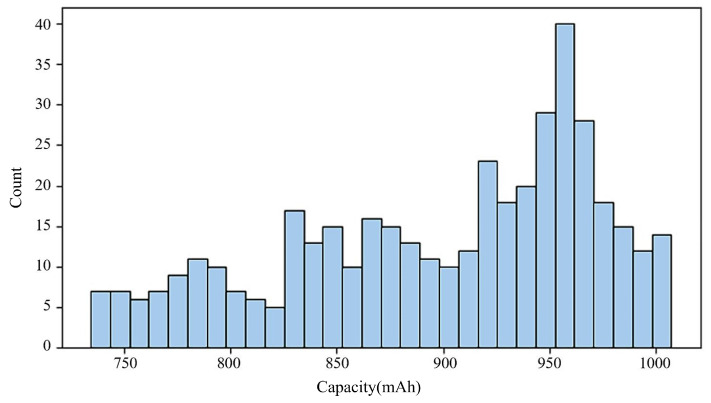
RUL-related feature histograms: capacity (mAh).

The existence of strong correlations with these performance metrics implies that these characteristics are essential for estimating the battery’s remaining lifespan. A visual representation of the relationship between the RUL and three selected features is provided by the histograms in Figure 6, Figure 7 and Figure 8. These visual distributions serve as an initial exploratory analysis of the data, establishing a basis for subsequent statistical analyses that will quantify the precise relationships between each feature and the RUL. They can be used as inputs to predict RUL, as variations in capacity, voltage decrement time, and discharge time are directly correlated with the health of a battery or RUL.

Abnormal data are eliminated using data cleansing and min–max normalization to enhance training, as it preserves the original data distribution, aside from a scaling factor, and converts all data to the range of [0, 1]. The dataset is partitioned into training and test sets in an 80:20 ratio, respectively [21]:

(4) $$x_{\mathrm{scaled}}=\frac{x-x_{\min}}{x_{\max}-x_{\min}}.$$

### 2.2. Feedforward Neural Network (FFNN)

**Figure 9:**
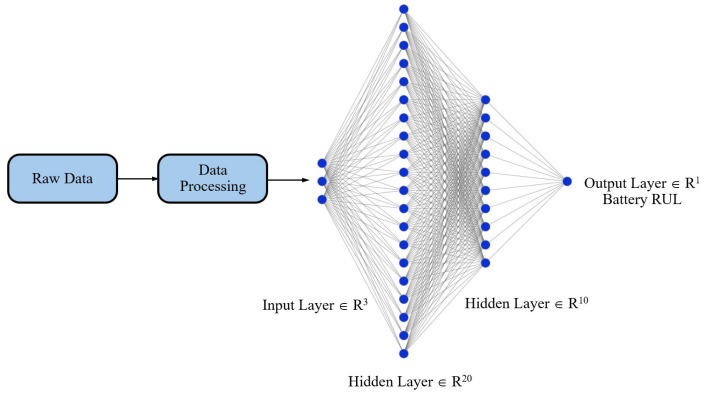
Feedforward neural network architecture.

To accurately estimate the remaining useful life (RUL) of lithium-ion batteries, we employed a feedforward neural network (FFNN) architecture designed to leverage three critical battery performance parameters—discharge time (s), decrement time within the 3.6–3.4 V interval (s), and capacity (mAh)—which are selected for their well-established correlation with cycle aging in lithium-polymer cells. As shown in Figure 9, the FFNN consists of an input layer with three neurons, two hidden layers that capture the nonlinear relationships among these features, and an output layer that yields the scalar RUL prediction. This model was specifically chosen to align with the practical constraints of the application, namely implementation on a resource limited embedded platform, i.e., the Raspberry Pi RP2040 microcontroller, built around a dual-core Arm Cortex-M0+. The FFNN’s architecture is inherently well suited for TinyML applications due to its low computational and memory footprint relative to more complex recurrent networks. This lightweight nature enables real-time inference to be performed directly on the embedded hardware, without reliance on cloud-based resources or specialized hardware accelerators. Moreover, the simplicity of the FFNN structure facilitates straightforward deployment and optimization, which is essential for ensuring low-cost, low-power, and low-latency operation in industrial battery management system applications [22].

The typical FFNN can be mathematically represented as

(5) $$y_{i}=\varphi_{0}\left[C\varphi_{h}\left(Bu_{i}+b_{h}\right)+b_{0}\right],$$

where $u_{i}\in R^{3}$ is the input vector at cycle *i*, containing the discharge time, decrement time, and capacity. Matrices *B* and *C* denote the learned weight matrices connecting the input layer to the hidden layers and the hidden layers to the output layer, respectively. Bias vectors $b_{h}$ and $b_{o}$ account for the biases in the hidden and output layers. Activation functions $\varphi_{h}$ and $\varphi_{o}$ introduce nonlinearity and enable the network to approximate complex mappings between input parameters and the battery’s RUL. $y_{i}\in R^{1}$ is the predicted remaining useful life (RUL) of the battery at cycle index *i*.

The rectified linear unit (ReLU) activation function in (6) optimizes and classifies distribution over output classes:

(6) $$f\left(x\right)=\max\left(0,x\right)=\left\{\begin{array}{cc} 0 & \mathrm{for}\;x\leq 0\\ x & \mathrm{for}\;x>0 \end{array}\right.,$$

During the training process, feedforward neural network models are constrained to minimize the error represented by the mean squared error (*MSE*) cost function, which may be stated as [23]

(7) $$MSE=\frac{1}{N}\sum_{k=1}^{N}{\left(y_{k}-{\hat{y}}_{k}\right)}^{2},$$

(8) $$J\left(\theta \right)=\frac{1}{2}\;E_{x,y∼{\hat{p}}_{\mathrm{data}}}{∥y_{k}-{\hat{y}}_{k}∥}_{2}^{2}+\mathrm{const},$$

where *N* represents the number of training input–output pairings, where ${\hat{y}}_{k}$ denotes the real output and $y_{k}$ signifies the model output. Equation (8) represents the loss function J(θ), which measures the discrepancy between the model’s predictions and the true values within the dataset. Specifically, it calculates the expected mean squared error (*MSE*) across all input–output pairs (x,y) sampled from the empirical data distribution ${\hat{p}}_{\mathrm{data}}$. The term $y_{k}-{\hat{y}}_{k}$ denotes the difference between the predicted output $y_{k}$ for the *k*-th sample and its corresponding actual RUL ${\hat{y}}_{k}$, with the squared Euclidean norm ${∥\cdot ∥}_{2}^{2}$ quantifying the magnitude of this error. The factor $\frac{1}{2}$ simplifies the computation of gradients during optimization. Finally, the constant term does not influence the gradient-based updates but may arise from certain normalization or regularization processes. Overall, this formulation ensures that the training procedure minimizes the average prediction error, thereby aligning the model’s parameters θ to improve accuracy.

### 2.3. TinyML On-Device Neural Network Training

TinyML is a machine learning technique that integrates optimized machine learning and embedded systems for performing on-device microcontroller analytics with low costs, less energy, and low latency [24]. TinyML is broadly expanded as a wide range of intelligent devices or applications, such as speech recognition mobile applications, object image recognition, intelligent early warning, and preventive maintenance systems. This research estimates the remaining useful life of the battery using the TinyML framework implemented on the Edge Impulse platform, facilitating efficient feature extraction, neural network algorithm design, machine learning model training, validation, testing, and optimization for deployment on embedded devices [25].

Edge Impulse is utilized to establish a pipeline for forecasting the remaining useful life (RUL) of a 3.7 V Li-Po battery. The model takes raw data as input and produces a scalar value ranging from 0 to 253, indicating the remaining useful life cycles. The training is set to 1000 cycles, with a learning rate of 0.005, and utilizes the CPU for processing. Furthermore, 20% of the dataset is designated for validation to evaluate model performance, and a batch size of 32 is established to optimize computational efficiency and memory utilization. The model is additionally refined for edge deployment via int8 quantization, minimizing its memory and processing requirements. The neural network architecture seen in Figure 9 is constructed via the Keras Sequential API, enabling the sequential stacking of layers. The architecture starts with two fully connected dense levels. The first dense layer comprises 20 neurons and utilizes the ReLU activation function, enabling the model to account for nonlinearity and enhance gradient flow during backpropagation. The subsequent dense layer comprises 10 neurons, employing ReLU activation. These layers facilitate the network’s acquisition of intricate patterns in the data by adding learned weights to the input and propagating it through the network. The final output layer is configured to provide a singular class, rendering this architecture appropriate for forecasting the remaining useful life (RUL) of a battery.

### 2.4. Model Performance Evaluation

The performance of the trained FFNN model will be evaluated using the testing dataset, which consists of battery data not utilized in the training or validation phases. Essential performance indicators will be computed to assess the precision and reliability of the RUL estimates [26]:

- Root Mean Squared Error: Assesses the average deviation between the predicted values and the actual values of RUL. A lower *RMSE* indicates higher prediction accuracy.
  (9) $$RMSE=\sqrt{\frac{\sum_{k=1}^{N}{\left(y_{k}-{\hat{y}}_{k}\right)}^{2}}{N}},$$
- Mean Absolute Error: Indicates the average absolute discrepancy between the predicted and actual remaining useful life values. A reduced *MAE* indicates superior predictive performance.
  (10) $$MAE=\frac{1}{N}\sum_{k=1}^{N}\left|y_{k}-{\hat{y}}_{k}\right|,$$
- R-squared: An elevated $R^{2}$ value indicates a superior alignment of the model with the data.
  (11) $$R^{2}=1-\frac{\sum_{k=1}^{N}{\left({\hat{y}}_{k}-y_{k}\right)}^{2}}{\sum_{k=1}^{N}{\left(y_{k}-\bar{y}\right)}^{2}},$$

## 3. Embedded AI Sensor for Real-Time RUL Estimation of UAV Batteries

### 3.1. Hardware Deployment

**Figure 10:**
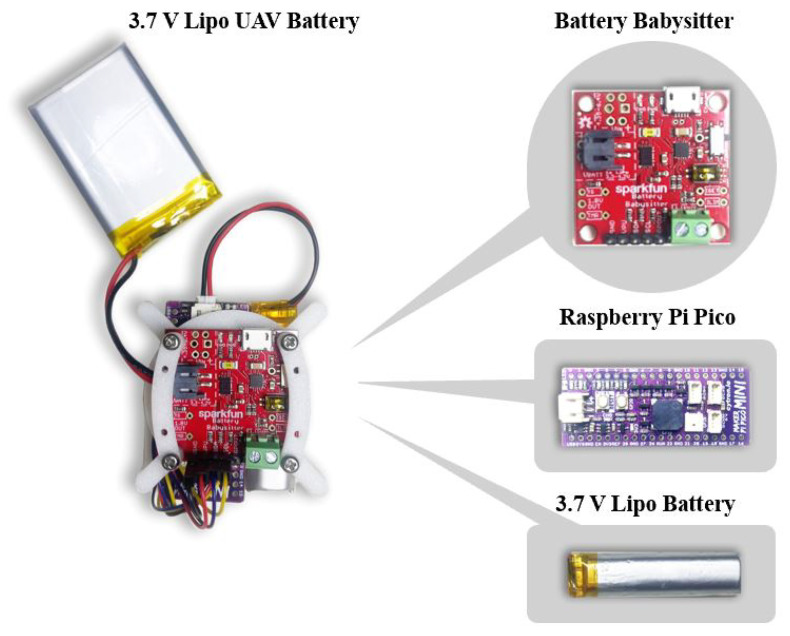
Embedded AI sensor components.

To manage and predict the remaining useful life (RUL) of a 3.7 V LiPo drone battery, an embedded AI sensor, shown in Figure 10, was developed using the SparkFun Battery Babysitter integrated with a Raspberry Pi Pico RP2040 microcontroller. The Battery Babysitter is a versatile battery management system designed for monitoring, charging, and protecting single-cell lithium-polymer (LiPo) batteries with a nominal voltage of 3.7 V. It combines multiple functionalities into a compact module, including charging, discharging, and power path management. The onboard charger, powered by the MCP73871 IC, allows the battery to charge via USB or external sources while simultaneously powering the load. It also features overcurrent, overvoltage, and undervoltage protection to ensure safe operation. The module includes I2C communication capabilities for real-time monitoring of battery parameters such as voltage, current, and charge–discharge status.

The Raspberry Pi Pico enables real-time measurement of key battery parameters through I2C communication, facilitating accurate RUL estimation using a trained TinyML model. Additionally, the microcontroller supports Bluetooth communication, allowing wireless data transfer and visualization on a laptop application. The data collected from the Battery Babysitter were processed using machine learning algorithms on the Raspberry Pi Pico, predicting battery remaining useful life based on observed discharge time and voltage decay time and capacity. This integrated system provides a comprehensive and efficient solution for battery monitoring and RUL estimation. The custom-developed LabVIEW application on the laptop was structured using state machine architecture to manage the reception, processing, and visualization of RUL data transmitted via Bluetooth BLE from the UAV.

**Figure 11:**
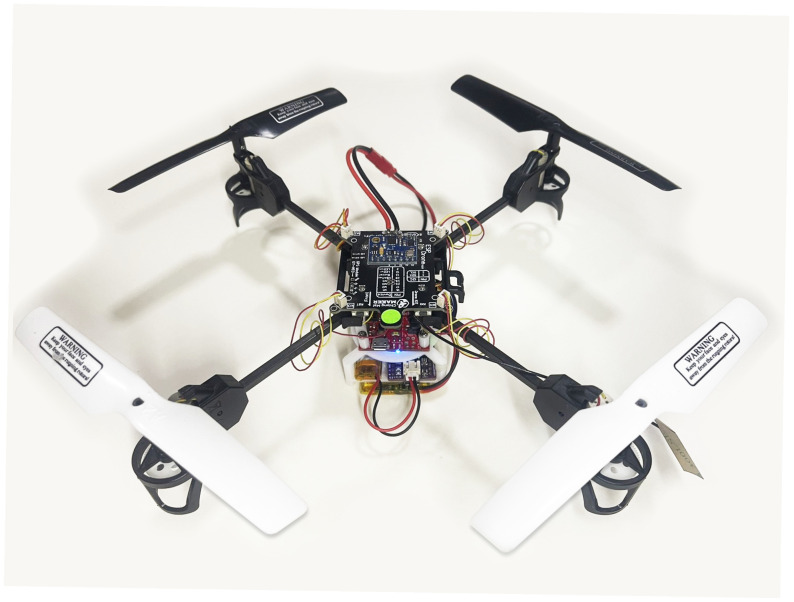
A quad-rotor UAV with RUL LiPo battery-embedded AI sensor.

The drone depicted in Figure 11 is a quad-rotor UAV equipped with the ESP32-S2 Wi-Fi module, facilitating remote control via a mobile application. The main control board is equipped with necessary sensors for core flight operations, including altitude and stability control. The quad-rotor arrangement utilizing 8520 DC motors and 140 mm CW/CCW propellers ensures efficient propulsion, with gear reduction devices enhancing torque while decreasing the propeller’s revolutions per minute (RPM).

### 3.2. Software Development

**Figure 12:**
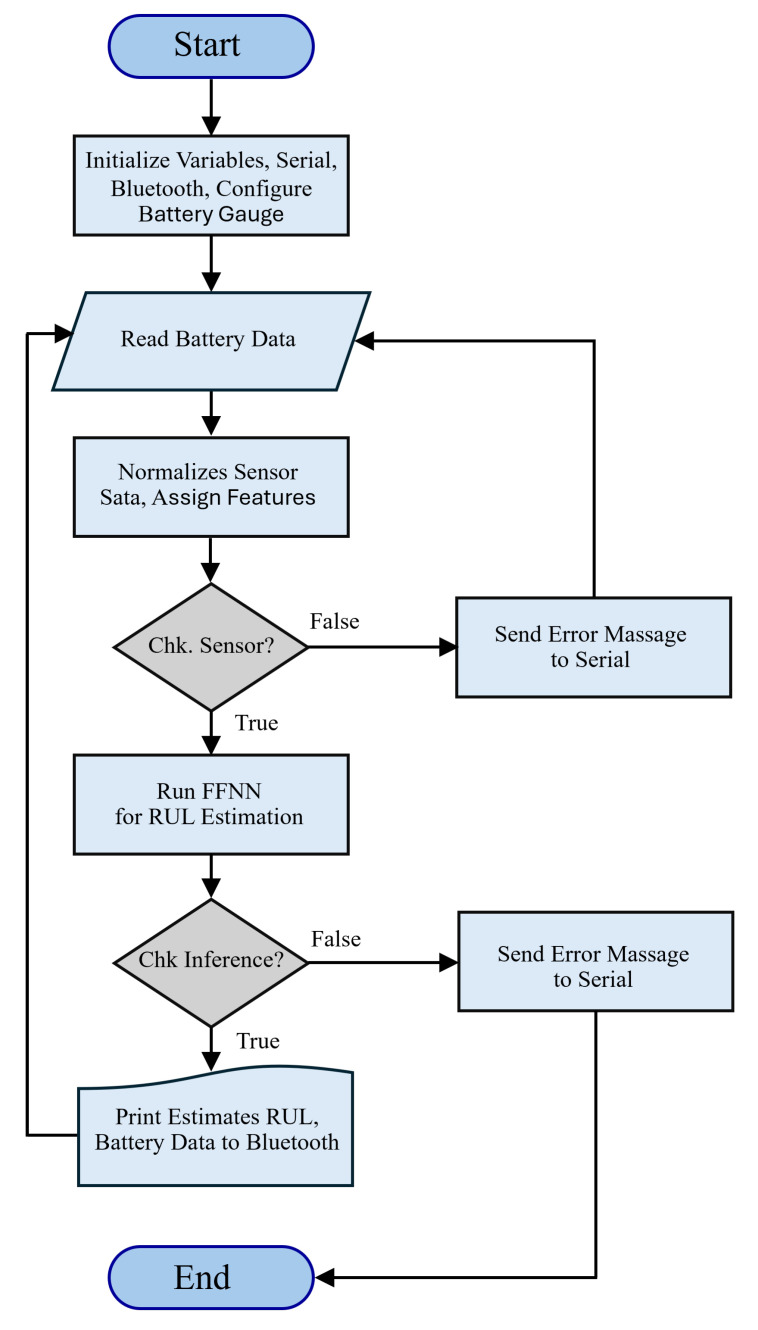
Flowchart of C/C++ code on embedded AI sensor.

The flowchart in Figure 12 illustrates the process of a C/C++ implementation on a Raspberry Pi Pico RP2040 W, which initializes and manages a battery monitoring system using a Babysitter BQ27441 battery gauge integrated with Edge Impulse’s TinyML framework to estimate the battery’s remaining useful life (RUL) using three features. The system starts by initializing variables, serial communication, Bluetooth, and the battery gauge configuration. It then reads battery data and normalizes the sensor data to assign relevant features for the RUL estimation model. If the sensor data pass a validation check, the FFNN model runs the RUL estimation process. The model inference is further checked for successful execution, and if all conditions are met, the estimated RUL and battery data are transmitted via Bluetooth to the LabView application on the laptop. Any failure in sensor validation or inference triggers an error message to be sent through the serial interface.

**Figure 13:**
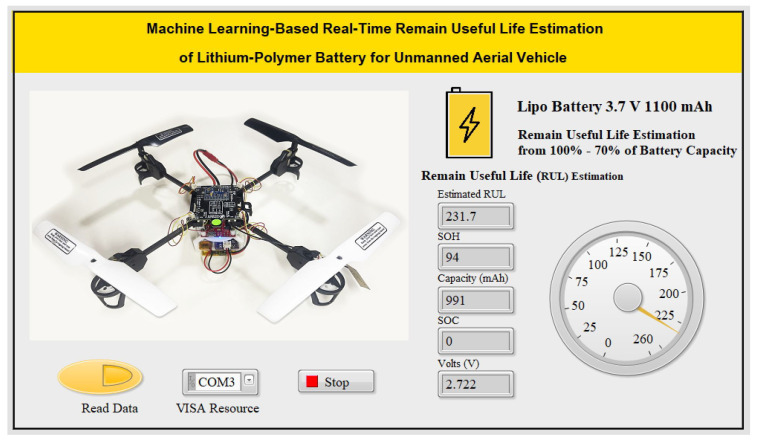
RUL LabView application software.

The graphical user interface (GUI) of the LabVIEW application in Figure 13 provides intuitive controls and displays for users. It includes buttons for starting/stopping data collection, configuring VISA resources for Bluetooth serial communication, and displaying battery parameters such as state of charge (SOC), state of health (SOH), capacity and the estimated RUL. The GUI ensures ease of interaction and efficient monitoring of RUL and battery performance in real time.

**Figure 14:**
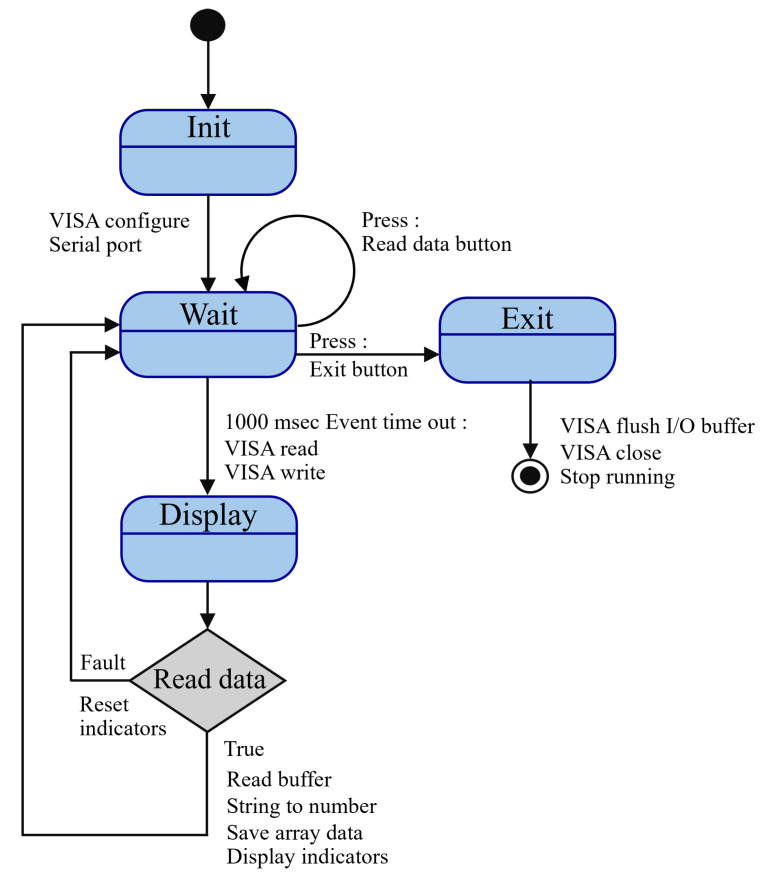
RUL LabView events trigger state machine diagram.

The application architecture is designed as a state machine [27,28]. As depicted in Figure 14, the process begins in the “Init” state, during which the system configures the serial port for communication via the VISA protocol. The program subsequently enters the “Wait” state, during which it monitors for input from users. Upon activating the “Read Data” button, the application transitions to the “Read Data” state, where it acquires battery metrics from the hardware through Bluetooth communication, processes the data (e.g., converting it from strings to numerical values), and verifies for any faults, resetting indicators as required. The data are subsequently transmitted to the “Display” state, where real-time battery metrics, including RUL and SOH, are refreshed on the GUI. The “Exit” state manages the application’s termination upon the user’s activation of the exit button. It ensures appropriate cleanup by flushing the I/O buffer, closing the VISA connection, and stopping the program. The modular state machine architecture facilitates explicit event management and logical advancement, guaranteeing the system effectively handles duties such as serial connection, data processing, and real-time display. This design is scalable and robust, rendering it ideal for battery monitoring and remaining useful life estimation in UAVs.

**Figure 15:**
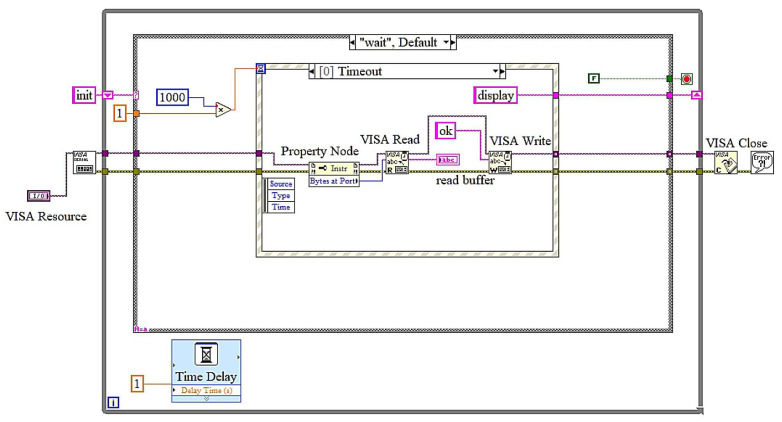
LabView block diagrams: wait state and timeout event.

**Figure 16:**
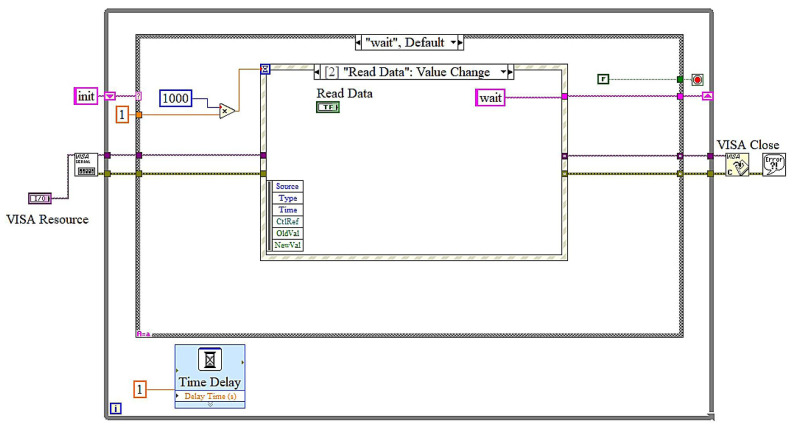
LabView block diagrams: wait state and read data event.

The LabVIEW block diagrams depict a state machine-based implementation for a real-time battery monitoring application, emphasizing remaining useful life (RUL) estimation. In the initialization state, the VISA protocol is configured to facilitate serial Bluetooth communication with the hardware. This state ensures the initialization of all requisite parameters, including baud rate and port configurations, so preparing the system for seamless operation. The wait state manages event-driven functions. The timeout event in Figure 15 ensures periodic data verifications, during which the system executes operations, including reading from and writing to the serial buffer every 1000 milliseconds. The read data event in Figure 16 is triggered when the user presses the “Read Data” button.

**Figure 17:**
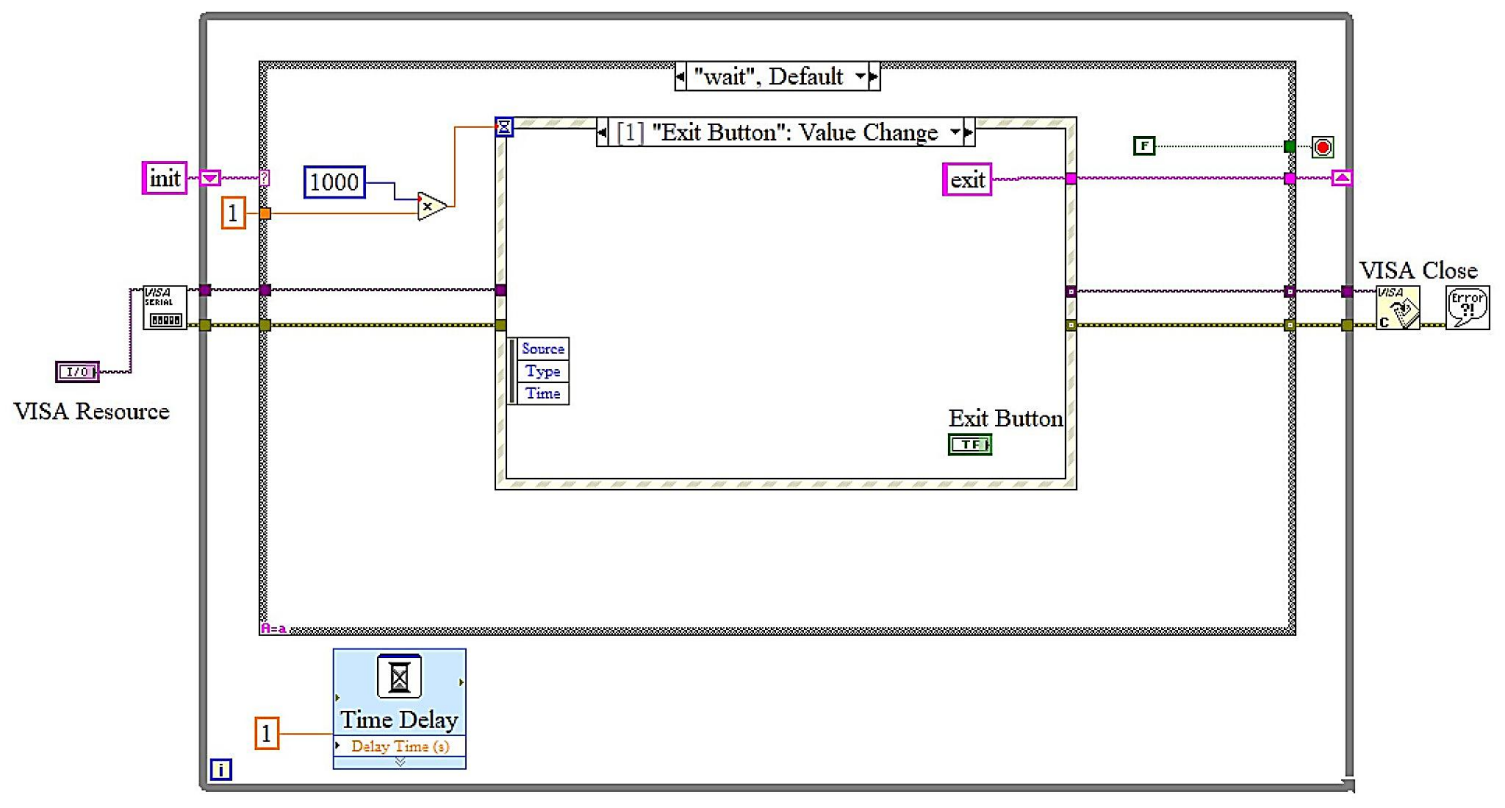
LabView block diagrams: wait state and exit event.

**Figure 18:**
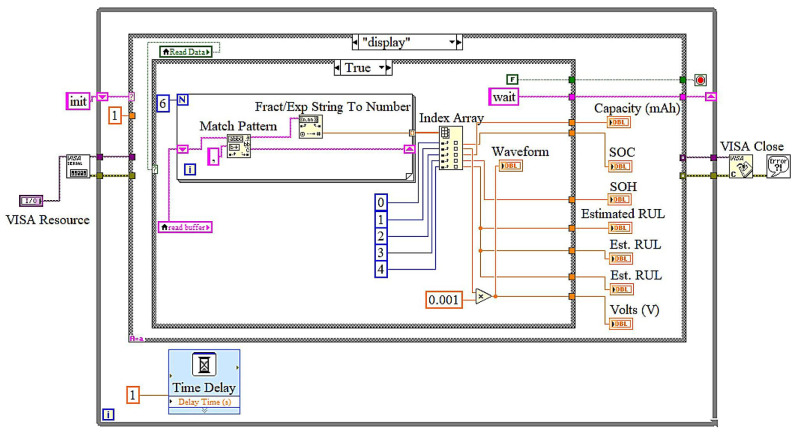
LabView block diagrams: display state and read data event.

During this event, data are extracted from the serial buffer, transformed into a usable format, and prepared for display or additional analysis. The exit event in Figure 17 is activated when the user presses the “Exit” button, initiating a systematic termination procedure. This involves flushing the I/O buffer, closing the VISA connection, and configuring the system for shutdown, ensuring no residual tasks persist. In the display state in Figure 18, the processed data, including battery capacity, state of health (SOH), and remaining useful life (RUL), are refreshed on the graphical user interface, offering real-time feedback to the user. Ultimately, the exit state ensures systematic termination of the application by executing cleanup operations, including shutting all processes and closing resources. The LabVIEW application, integrating an embedded AI sensor, offers a reliable and effective solution for real-time remaining useful life assessment of lithium-polymer batteries. The system guarantees modularity and reliability by employing a state machine architecture with clearly specified states. The user-friendly interface facilitates effortless interaction, while real-time data visualization provides precise monitoring of essential battery parameters.

## 4. Results and Discussion

### 4.1. Model Training and Testing Results

**Figure 19:**
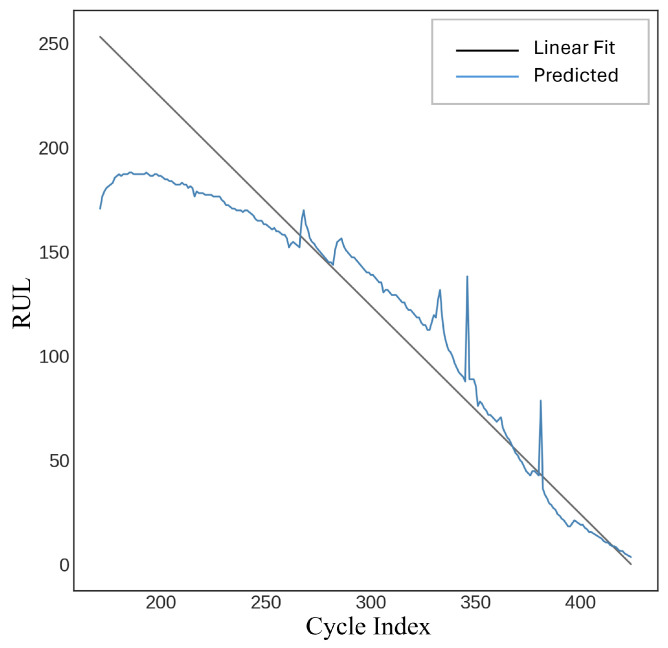
Feedforward neural network (FFNN).

A feedforward neural network (FFNN) employing a multi-layer perceptron regressor was utilized to estimate a target remaining useful life (RUL) variable. The model is defined with specific hyperparameters, such as a ReLU activation function, an Adam optimizer for enhancement, and multiple hidden layers. The training procedure is time-constrained, and predictions are generated on the test data ($X_{\mathrm{test}}$) after the model has been fitted to the training data ($X_{\mathrm{train}},\;Y_{\mathrm{train}}$). Performance metrics, including R-squared, root mean squared error (*RMSE*), and mean absolute error (*MAE*), are computed and documented. The model attained an R-squared value of 89.14%, an *RMSE* of 24.17, and an *MAE* of 17.62, as illustrated in Figure 19.

### 4.2. TinyML FFNN Neural Network Optimized Model Training and Testing Results

#### 4.2.1. FFNN Neural Network Model Optimization

In this study, five optimized TinyML feedforward neural network (FFNN) models were trained and evaluated for a battery remaining useful life (RUL) prediction task, with a focus on model size, accuracy, and computational efficiency. Model 1, with a simple structure of Dense(20) → Dense(10) layers using only three input features, achieved the best overall performance, recording a loss of 57.24, a mean absolute error (*MAE*) of 5.83, and an explained variance score of 0.99. It also demonstrated the lowest memory usage (1.2 kB RAM, 11.0 kB flash) and fastest inference time (2 ms), making it highly suitable for resource-constrained devices like the Raspberry Pi RP2040. Models 2 and 5, which employed a larger architecture with Dense(40) → Dense(20) neurons and dropout regularization, achieved similar variance scores but slightly higher memory consumption (11.8 kB flash). Model 4 exhibited slightly worse performance with a higher loss (70.00), while Model 3 performed poorly overall with a loss exceeding 14,000 and was deemed unsuitable for deployment.

Based on the comparative results in Table 3, Model 1 is recommended for deployment in embedded TinyML systems due to its optimal balance of accuracy, size, and speed. Its minimal resource demands and robust predictive ability make it ideal for real-time RUL estimation on ultra-low-power platforms. In contrast, Model 3 should be discarded due to ineffective learning and inefficient memory use. Future work may involve fine-tuning hyperparameters and applying advanced feature engineering techniques to further enhance performance while maintaining the lightweight profile essential for embedded applications.

**Figure 20:**
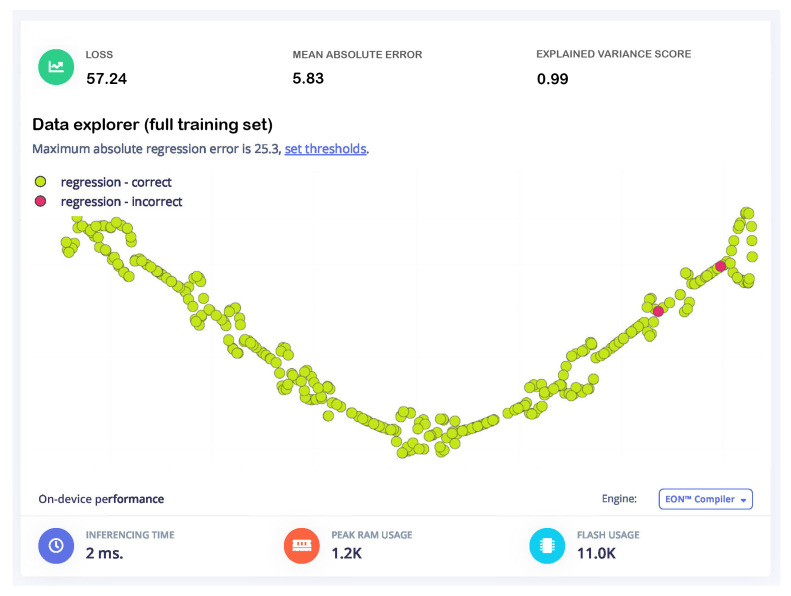
Training performance and data explorer.

Figure 20 illustrates the results of training a feedforward neural network (FFNN) model with the Edge Impulse platform. The degree of variation between expected and actual values is reflected in the key metrics of a mean squared error (*MSE*) of 57.24 and a mean absolute error (*MAE*) of 5.83. The explained variance score of 0.99 indicates the model’s remarkable ability to account for data variability, underscoring its robust predictive efficacy. The training loss is recorded at 57.24, corresponding with the *MSE*, indicating consistent model convergence. Green dots indicate correct regression predictions and red dots indicate faulty predictions based on a threshold (maximum absolute regression error of 25.3) in the graphical data explorer. The model is efficient for embedded devices with an inference time of 2 ms, peak RAM consumption of 1.2 KB, and flash memory utilization of 11 KB. Ideal for real-time applications, this compact and quantized model is accurate and resource-efficient.

**Figure 21:**
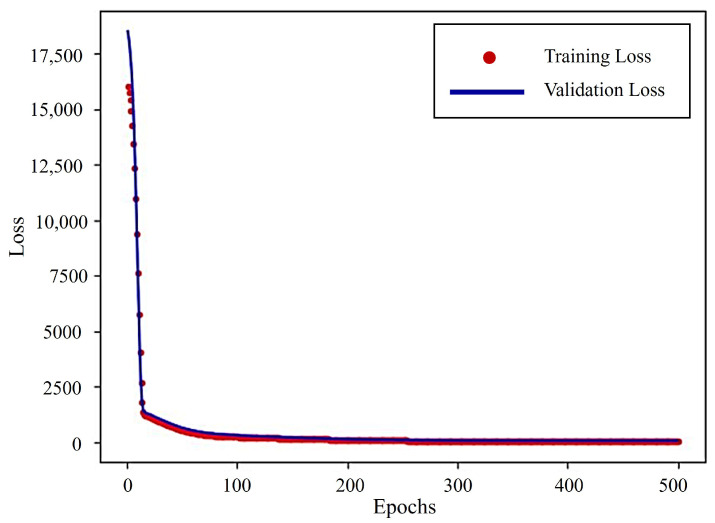
Training and validation loss.

The red spots denote the training loss, whereas the blue line indicates the validation loss in Figure 21. Initially, the loss values are very high, indicating significant errors in predictions at the start of training. However, both losses decrease rapidly during the early epochs, demonstrating effective learning by the model. Around 500 epochs, the loss values plateau, reaching a consistent and low range, suggesting convergence of the model. The model’s ability to generalize well with minimal overfitting is suggested by the close alignment of the training and validation loss curves.

#### 4.2.2. FFNN Neural Network Testing Results

**Figure 22:**
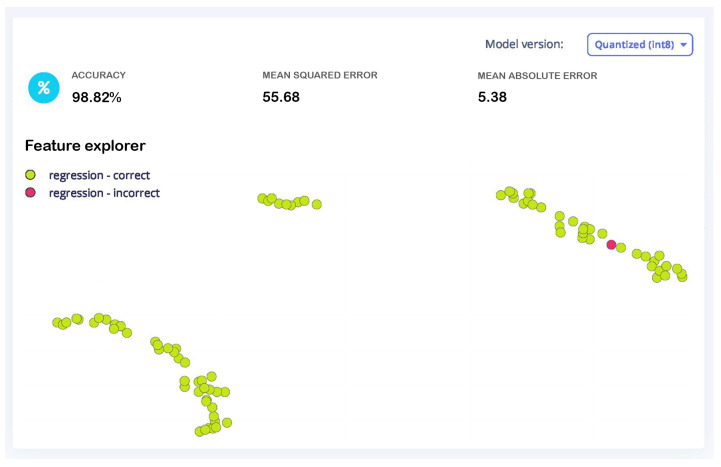
Testing result data explorer.

**Figure 23:**
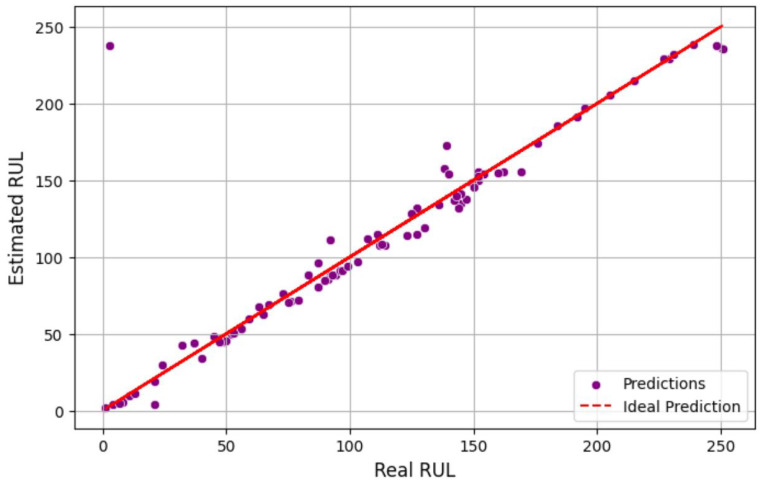
Scatter plot of estimated RUL testing results.

This performance underscores the model’s capacity to perform consistently on unseen data and the efficacy of the training process. Figure 22 displays the testing results of a machine learning regression model, with the model version specified as quantized (int8). Figure 23 illustrates the comparison between the actual remaining useful life (RUL) and the estimated RUL from the model testing. In the scatter diagram, the relationship between the estimated RUL values and the actual RUL values is illustrated. The red dashed line represents the ideal prediction where the estimated RUL matches the real RUL perfectly. The proximity of data points to the red line indicates the accuracy of the prediction model. The model testing results indicate an overall accuracy of 98.82%, alongside a mean squared error (*MSE*) of 55.68 and a mean absolute error (*MAE*) of 5.38.

Table 4 presents the deployment testing results for a TinyML model optimized for real-time applications. Two model versions are compared: a quantized (int8) version and an unoptimized (float32) version, both deployable via the Arduino library and the EON™ Compiler on ARM-based embedded platforms. While both models exhibit identical accuracy at 98.82% and consume the same amount of RAM (1.2 KB), the quantized model achieves a significantly lower inference latency of 2.0 ms compared to 10.0 ms for the unoptimized counterpart. Flash memory usage is also comparable, with 11.0 KB for the quantized model and 10.7 KB for the unoptimized version. These findings highlight the primary benefit of quantization in latency reduction, making the quantized model more suitable for time-sensitive, resource-constrained embedded systems. The results confirm the effectiveness of the EON™ Compiler in delivering high-performance, low-latency models without sacrificing predictive accuracy.

### 4.3. Embedded AI Sensor for Real-Time RUL Estimation Results

**Figure 24:**
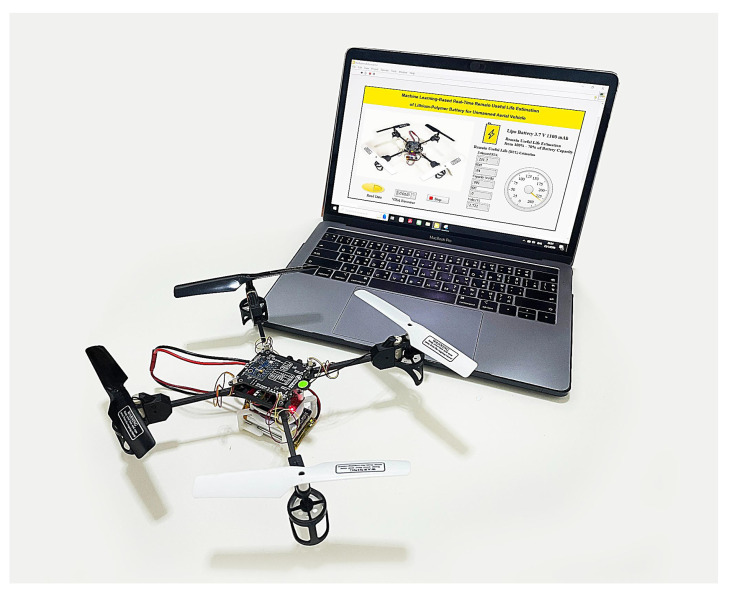
RUL estimation experiment.

Figure 24 illustrates the experimental setup and results of the remaining useful life (RUL) estimation system for a lithium-polymer (LiPo) battery used in an unmanned aerial vehicle (UAV). The image shows a functional UAV equipped with a Raspberry Pi RP2040 microcontroller, which processes real-time battery sensor data and predicts the RUL using an embedded feedforward neural network (FFNN) model. The predictions are transmitted via Bluetooth to a laptop running a LabVIEW application with a state machine architecture. The LabVIEW interface displays key battery parameters such as capacity, state of health (SOH), and RUL in real time, offering a user-friendly visualization of the battery’s performance. Validation experiments were conducted on an 80 g UAV powered by a 1100 mAh LiPo battery. The results were obtained using the input features processed on a Raspberry Pi Pico RP2040 W, running a trained FFNN (feedforward neural network) model.

In this study, the remaining useful life (RUL) of the UAV’s LiPo battery is estimated during in-flight operation, following a full charge and UAV takeoff. The embedded AI module continuously acquires real-time data on battery capacity, discharge time, and voltage decay time throughout the flight mission. Notably, RUL estimation is conducted in real time rather than post-operation, enabling immediate insight into battery health. It is important to emphasize that the predicted RUL values are considered valid only within the operating voltage range of 2.70 V to 2.799 V, which has been empirically validated and is traceable within the LabVIEW-based monitoring environment. This constraint ensures both the accuracy and operational reliability of the RUL predictions under embedded deployment conditions.

The feedforward neural network (FFNN) model demonstrates exceptional accuracy and consistency in estimating the remaining useful life (RUL) of UAV LiPo batteries under real-time conditions obtained from the LabVIEW-based monitoring system, as presented in Table 5. The experimental setup involved two distinct LiPo batteries (Battery 1 and 2), representing high and medium RUL ranges, respectively. The model achieved a mean absolute error (*MAE*) of 3.46 cycles, a root mean squared error (*RMSE*) of 3.75 cycles, and a coefficient of determination ($R^{2}$) of 0.9977. The average prediction error of 0.80 cycles indicates a slightly conservative estimation, which is generally favorable in safety-critical applications. These accuracy metrics from the experimental results are marginally superior to those obtained from the model testing phase. It is important to note that the experimental results were derived from only 30 real-time data points, while the model testing results were based on a broader test dataset comprising 20% of the entire dataset, which spans a wider RUL range.

### 4.4. Performance Comparison with Related Work

**Figure 25:**
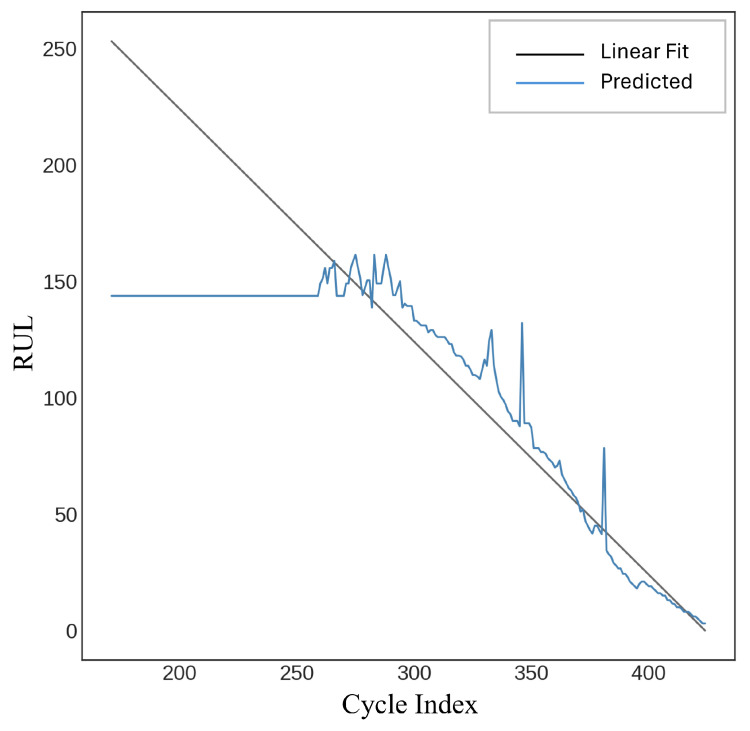
K-nearest neighbors (KNNs).

The K-Nearest Neighbor (KNN) Regressor was employed to predict a remaining useful life (RUL) variable [29]. The model is initialized with n_neighbors set to 3, indicating that it takes into account the three closest neighbors for regression. Performance metrics, including R-squared, root mean squared error (*RMSE*), and mean absolute error (*MAE*), are computed for evaluation after predictions are generated using test data (X_test_). The measurements, accompanied by temporal information, are recorded in a DataFrame. The model achieved an R-squared score of 66.16%, with a root mean square error (*RMSE*) of 42.65 and a mean absolute error (*MAE*) of 29.04, as illustrated in Figure 25.

**Figure 26:**
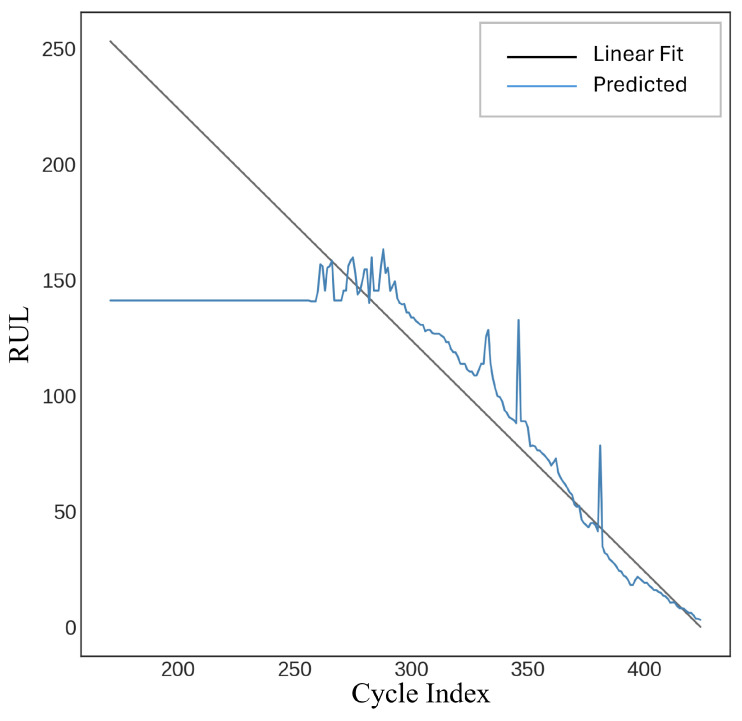
Random Forest.

The objective remaining useful life (RUL) variable was forecasted using the Random Forest Regressor [30]. The model is initialized using many hyperparameters: n _estimators = 100 (indicating 100 decision trees), max _features =’sqrt’, and more configurations, including bootstrap = True, to utilize bootstrapped datasets. The model is trained using X_train_ and Y_train_, and the training duration is documented. Using the test data (X_test_), predictions are generated, and evaluation metrics such as R-squared, root mean squared error (*RMSE*), and mean absolute error (*MAE*) are calculated. Figure 26 presents an R-squared score of 63.62%, an *RMSE* of 44.22, and an *MAE* of 30.08.

**Figure 27:**
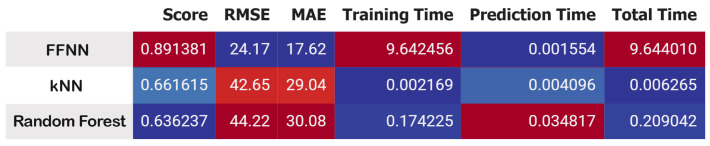
RUL estimation performance comparison.

Figure 27 presents a comparison matrix assessing three models, FFNN, KNN, and Random Forest, according to their scores (R-squared), *RMSE*, *MAE*, and computational times. FFNN has superior performance with an R-squared of 0.8913, the minimal *RMSE* of 24.17, and the lowest *MAE* of 17.62, establishing it as the most precise model. Nonetheless, its training duration is considerably greater at 9.64 seconds, rendering it less computationally efficient. KNN exhibits middling performance, attaining an R-squared value of 0.6616, although it is the most rapid in both training and prediction, with an overall runtime of merely 0.0063 seconds. Random Forest exhibits a little reduced R-squared value (0.6362) relative to KNN, alongside the greatest *RMSE* (44.22) and *MAE* (30.08) with an overall runtime of 0.209 seconds. In summary, FFNN excels in accuracy, whereas KNN is superior in speed.

The selection of FFNN, Random Forest Regressor, and kNN was intended to provide a balanced comparison between neural and non-neural models with differing computational complexities. FFNN was chosen for its simplicity and suitability for deployment on embedded platforms with limited resources, such as the Raspberry Pi RP2040. In contrast, Random Forest and kNN serve as robust, widely used non-deep learning baselines. This combination enables a meaningful evaluation of trade-offs between model accuracy and deployment feasibility in real-world battery management applications.

### 4.5. Challenges and Potential Improvements

While the proposed TinyML-based framework demonstrates promising real-time RUL estimation performance on a resource-constrained embedded platform, several challenges remain. The use of a compact feedforward neural network (FFNN) was necessary to accommodate the strict memory and computational limitations of the Raspberry Pi RP2040 microcontroller. However, more sophisticated architectures, such as long short-term memory (LSTM) networks, could potentially capture temporal dependencies in degradation patterns more effectively, offering improved prediction accuracy. The current hardware platform’s limited RAM, flash storage, and processing capabilities restrict the deployment of such complex models. Future work may explore the integration of optimized LSTM variants, model compression techniques, or hardware accelerators to overcome these limitations. Additionally, advancements in lightweight neural architectures specifically tailored for TinyML applications could further enhance the precision and robustness of real-time RUL prediction systems for UAV battery monitoring.

## 5. Conclusions

This study proposes an embedded TinyML-based framework for real-time estimation of the remaining useful life (RUL) of lithium-polymer (LiPo) batteries in unmanned aerial vehicles (UAVs). By integrating sensor data fusion with a lightweight feedforward neural network (FFNN) model optimized via the Edge Impulse platform, the system successfully addresses the computational constraints of low-power embedded devices. Experimental evaluations conducted on an 80-gram UAV equipped with a 1100 mAh LiPo battery demonstrate that the proposed framework achieves a mean absolute error (*MAE*) of 3.46 cycles and a root mean squared error (*RMSE*) of 3.75 cycles. Model testing further confirms the robustness and precision of the approach, yielding an overall accuracy of 98.82%, a mean squared error (*MSE*) of 55.68, a mean absolute error (*MAE*) of 5.38, and a variance score of 0.99. Regarding embedded deployment performance, the quantized (int8) version of the model achieves an inference latency of just 2 ms, with memory utilization limited to 1.2 KB RAM and 11 KB flash, highlighting its suitability for real-time RUL estimation on resource-constrained microcontrollers such as the Raspberry Pi RP2040. Overall, the results validate the feasibility and effectiveness of combining embedded sensor data fusion and TinyML techniques to enable accurate, low-latency, and resource-efficient RUL prediction for UAV battery health monitoring. Future work will aim to enhance the framework further by investigating advanced lightweight architectures, such as optimized LSTM networks, to improve temporal feature extraction under embedded system limitations.

## Figures and Tables

**Table 1:** Charging and discharging profiles associated with the collected battery degradation data.

	Charge	Discharge
Profile	CC–CV	CC
	(Constant Current–Constant Voltage)	(Constant Current)
Charge Current	1.0 A	–
Voltage Threshold	4.2 V (CV phase)	Cut-off Voltage 2.75 V
Discharge Current	–	3.0 A
Temperature	25 °C	25 °C

**Table 2:** Battery features.

	Count	Mean	Std.	Min.	25%	50%	75%	Max.
Discharge Time (s)	424	1078.29	86.84	881.33	1013.34	1102.11	1148.43	1029.43
Decrement 3.6–3.4V (s)	424	199.77	57.31	83.99	147.99	210.0	238.99	281.0
Maximum Voltage Discharge (V)	424	3.82	0.02	3.77	3.80	3.82	3.84	3.85
Minimum Voltage Charge (V)	424	3.44	0.07	3.07	3.41	3.44	3.48	3.52
Time at 4.15V (s)	424	2370.30	333.15	3.38	2127.11	2454.43	2623.67	2947.42
Capacity (mAh)	424	898.27	72.36	734.07	844.06	918.0	956.59	1007.44
RUL	424	109.66	67.97	0	52.75	105.5	158.25	253.0

**Table 3:** Model structure and performance summary.

Model	Neural Network Architecture	Loss (*MSE*)	*MAE*	Variance	Latency	Peak RAM	Flash Usage
Model 1	Dense(20)–Dense(10)–Output(1)	57.24	5.83	0.99	2 ms	1.2 kB	11.0 kB
Model 2	Dense(40)–Dense(20)–Dropout(0.5)– Output(1)	57.59	5.77	0.99	2 ms	1.2 kB	11.8 kB
Model 3	Dense(20)–Dense(10)–Dense(5)– Dropout(0.25)–Output(1)	14,218	98.36	0.00	6 ms	1.4 kB	11.3 kB
Model 4	Dense(20)–Dense(10)–Dropout(0.25)– Output(1)	70.00	6.73	0.99	2 ms	1.2 kB	11.1 kB
Model 5	Dense(40)–Dense(20)–Dropout(0.5) –Output(1)	57.59	5.77	0.99	2 ms	1.2 kB	11.8 kB

**Table 4:** On-device performance comparison between quantized (int8) and unoptimized (float32) versions.

	Quantized (int8)	Unoptimized (float32)
Latency	2.0 ms	10.0 ms
RAM	1.2 K	1.2 K
Flash	11.0 K	10.7 K
Accuracy	98.82%	98.82%

**Table 5:** Real-time RUL estimation of UAV LiPo battery records.

Batt. No.	Test No.	Estimated RUL	Actual RUL	SOH (%)	SOC (%)	Volts (V)
1	1	231.7	231	94	0	2.722
1	2	228.86	230	94	0	2.720
1	3	226.96	229	94	0	2.728
1	4	226.01	228	94	0	2.721
1	5	225.06	227	94	0	2.725
1	6	223.16	226	94	0	2.727
1	7	220.31	225	94	0	2.723
1	8	217.46	224	94	0	2.720
1	9	216.51	223	93	0	2.726
1	10	214.61	222	93	0	2.733
2	1	78.82	74	83	0	2.775
2	2	77.52	73	83	0	2.766
2	3	75.97	72	83	0	2.732
2	4	74.07	71	83	0	2.745
2	5	73.12	70	83	0	2.748
2	6	72.17	69	83	0	2.721
2	7	71.22	68	83	0	2.719
2	8	70.27	67	82	0	2.778
2	9	69.32	66	82	0	2.793
2	10	68.37	65	82	0	2.765
2	11	54.13	57	81	0	2.760
2	12	53.18	56	81	0	2.788
2	13	52.23	55	80	0	2.751
2	14	51.28	54	80	0	2.723
2	15	50.33	53	80	0	2.773
2	16	49.38	52	80	0	2.798
2	17	47.48	51	80	0	2.739
2	18	45.57	50	80	0	2.758
2	19	44.63	49	79	0	2.734
2	20	51.28	48	79	0	2.747

## Data Availability

The raw data supporting the conclusions of this article will be made available by the authors on request.

## Abbreviations

The following abbreviations are used in this manuscript:

AI	Artificial Intelligence
CNNs	Convolutional Neural Networks
DCNNs	Deep Convolutional Neural Networks
DNNs	Deep Neural Networks
EON™	Energy-Efficient On-Device Inference Compiler
EV	Electric Vehicle
FDA	Functional Data Analysis
FFNN	Feedforward Neural Network
GUI	Graphical User Interface
IoT	Internet of Things
KNN	K-Nearest Neighbor
LiPo	Lithium-Polymer Battery
LTCNs	Lightweight Temporal Convolutional Networks
LSTM	Long Short-term Memory
NASA	National Aeronautics and Space Administration
*MAE*	Mean Absolute Error
*MSE*	Mean Squared Error
PF	Particle Filter
ReLU	Rectified Linear Unit
*RMSE*	Root Mean Squared Error
RUL	Remaining Useful Life
SOC	State of Charge
SOH	State of Health
UAV	Unmanned Aerial Vehicle
VISA	Virtual Instrument System Architecture
